# Clinical efficacy of 9-oxo-10, 11-dehydroageraphorone extracted from *Eupatorium adenophorum* against *Psoroptes cuniculi* in rabbits

**DOI:** 10.1186/s12917-014-0306-4

**Published:** 2014-12-20

**Authors:** Yang Hu, Fei Liao, Yanchun Hu, Biao Luo, Yajun He, Quan Mo, Zhicai Zuo, Zhihua Ren, Junliang Deng, Yahui Wei

**Affiliations:** Key laboratory of Animal Disease and Human Health of Sichuan Province, College of Veterinary, Medicine, Sichuan Agricultural University, Sichuan Province Ya an, 625014 China; Qiandongnan Prefectural Center for Animal Disease Control and Prevention of Guizhou province, Kaili, 556000 China; Key Laboratory of Resource Biology and Biotechnology in Western China, School of Life Science, Northwest University, Xi’an, 710069 China

**Keywords:** Euptox A, *Eupatorium adenophorum*, *Psoroptes cuniculi*, Acaricidal, Clinical efficacy

## Abstract

**Background:**

Animal acariasis is one of the important veterinary skin diseases. Chemical drugs have been widely used to treat and control this kind of disease. But many chemicals control could increase resistance in target species, toxicity and environmental hazards. We found that the 9-oxo-10, 11-dehydroageraphorone (euptox A) extracted from *E. adenophorum* has strong toxicity against *P. cuniculi* in vitro, but the in vivo acaricidal actions of euptox A have yet to be investigated.

**Results:**

A 14-day experiment was performed using rabbits that were naturally infested with *P. cuniculi* on a farm. Rabbits were randomly divided into five groups; animals in groups A, B and C were treated in each ear topically with 4.0 ml of 2.0 and 1.0 g/L (w/v) euptox A, respectively. Animals in groups D and E were treated with ivermectin (by injection; positive controls) and glycerol with water only (by embrocation; negative controls), respectively. Each rabbit was treated twice with separate treatments on days 0 and 7. Rabbits were observed daily and detailed examinations were performed on days 0, 7 and 14, to inspect the presence or absence of mites and scabs/crusts. Seven days after the initial treatment, the mean clinical scores (presence of scabs/crusts) decreased from 3.48, 3.37, 3.43 and 3.45 to 0.37, 0.42, 0.78 and 0.38 in the ears of animals in groups A, B , C and D, respectively, which were similar to the observations recorded in the positive control rabbits. However, the clinical score for negative control rabbits did not increase significantly (P > 0.05) during the experiment, and this changed from 3.32 to 3.37 in the ears, and there were no significant differences in clinical efficacy between left and right ears. After two treatments (0 and 7 d), the rabbits in groups A, B, C and D had recovered completely 14 days after the last treatment and no recurrences of infection were observed.

**Conclusions:**

These results indicate that euptox A was potent compounds for the effective control of animal *P. cuniculi* in vivo.

## Background

Animal acariasis, an important veterinary diseases, may reduce the productivity and the quality of animal products, even lead to death [1]. At present, chemical drugs are widely used to treat and control the *psoroptes* and *sarcoptic* mange in veterinary clinic, and obtained the relative good treatment effectiveness, including ivermectin, and abamectin, etc. But the chemical control could increase resistance in target species, toxicity and environmental hazards [2].

A large number of reports have indicated that the secondary metabolites synthesized and accumulated in *Eupatorium adenophorum* (*E. adenophorum*) have wide biological activities. For example, there are reports of chronic respiratory disease and exercise intolerance in horses in Australia due to ingestion of *E. adenophorum* [3]. The ethanol extract from leaves of *E. adenophorum* were anti-Inflammatory potential [4], acaricidal activity [5,6], antioxidant activity [7], and other extract form *E. adenophorum torium* had toxic activity against *Oncomelania hupensis*, the intermediate host snail of *Schistosoma japonicum* [8], *Tinea* [9] and *Aphis gossypii* [10]*.* The acetone extract of *E. adenophorum* had strong toxicity against Cabbage *aphids* and *Brevicoryne brassicae* [11].

9-oxo-10, 11-dehydroageraphorone (euptox A), is the main toxin extracted from *E. adenophorum* [12,13]. Euptox A takes a large proportion of *E. adenophorum* toxins [14,15], can cause not only the allergic bronchial pneumonia of horses which is characterized by pulmonary interstitial fibrosis, emphysema, alveolar epithelisation and reduced tolerance to exercise [3,16], but the contact dermatitis of other domestics animals like cattle and goats [17]. Furthermore, according to some studies, for mice, lesions occur in the liver. The hepatic injury in these animals is characterised by multiple areas of focal necrosis of the parenchyma associated with degeneration and loss of the epithelium lining the small bile ducts [18,19]. Euptox A belongs to a cadenine sesquiterpene. A large number of reports indicated that the cadenine sesquiterpene has wide biological activities such as antitumor activity [20], antigerminative activity [21], neurotrophic activity [22], larvicidal activity [23], antiprotozoal activity [24], and so on. Euptox A was found highly active against the fast growing A549, Hela and Hep-2, and its activity was concentration-dependent [25]. In a previous study, euptox A exhibited strong toxicity against *S. scabiei* and *P. cuniculi in vitro* [26], but the *in vivo* acaricidal actions of euptox A have yet to be investigated.

Thus, the aim of this present study was to assess the clinical acaricidal efficacy of euptox A against *P. cuniculi* in rabbits *in vivo*.

## Methods

### Extraction and purification of euptox A

*E. adenophorum* was collected from Xichang City (102°30222′E, 27°873213′S) of Sichuan Province, Southwest China in July, 2012. Euptox A was extraction from *E. adenophorum* by the Key laboratory of Animal Disease and Human Health of Sichuan Agricultural University, China [25-27].

### Experimental animals

Thirty rabbits that were naturally infected with *P.cuniculi* were obtained from a farm affected by an outbreak. All the animals were of similar age, weight and clinical score (in terms of the presence/absence of scabs and crusts). In all 30 rabbits, there were no significant difference between the level of infection in the ears, and had not been treated with acaricides and no other infectious diseases were known to be present. Sampling procedures adhered to institutional ethical and animal care guidelines and all methods were conducted in accordance with the Guide for the Care and Use of Laboratory Animals adopted and promulgated by the United National Institutes of health. All experimental protocols were approved by review committee for the use of human or animal subjects of College of veterinary medicine, Sichuan Agricultural University.

### Acaricidal activity *in vivo*

The euptox A was diluted from the concentration of 4 mg/ml to 1 mg/ml (4 mg/ml, 2 mg/ml and 1 mg/ml) in 10% glycerin.

The experimental procedures were performed as described previously by [28]. The thirty rabbits were divided into five equal groups at random (groups A, B, C, D and E). Groups A, B and C were treated at days 0 and 7 with 4, 2 and 1 mg/ml of euptox A, respectively. Groups D and E were treated twice with ivermectin (by injection 200 μg/kg) and glycerol and water only (by embrocation) as the positive and untreated control groups, respectively. In the treatment process, each of the left and right ears were treated with the use of a cotton swab dipped with 2 ml of the euptox A, then embrocated treatment as an ointment. The presence of clinical signs was assessed daily and any abnormal reactions were recorded. Detailed examinations were performed on days 0, 7 and 14 which included an assessment of the presence or absence of mites and an evaluation of clinical infection and the degree of recovery. The criteria used to evaluate the clinical infection score and degree of recovery can be found in Table 1 [28].

**Table 1:** **Parameters used to evaluate the clinical score of infection and degree of recovery**

**Infection and degree of recovery**	**Clinical score**
Absence of scabs and/or mites	0
Irritation in ear canal but no mites observed	0.5
Small number of scabs in the ear canal, mites present	1
External ear canal filled with scabs, mites present	2
Scabs in ear canal and proximal 1/4 of pinna, mites present	3
1/2 pinna filled with scabs, mites present	4
3/4 of the pinna filled with scabs, mites present	5
All internal surface of the pinna full of scabs, mites present	6

### Statistical analysis

Statistical analysis was performed using SPSS software (SPSS, version 20.0) [29] to assess the presence of statistically significant differences in infection and recovery scores, and therapeutic effects of different concentrations of euptox A and different treatment times. Significance values were corrected for with Duncan’s multiple comparisons test [30].

## Results

The criteria used to evaluate the clinical infection score and degree of recovery can be found in Table 1, The mean degree of infection was similar in each group at the start of the experiment and no significant differences were detected (Table 2).

**Table 2:** **The clinical score of infection and degree of the recovery from infection of the external ear margins in rabbits (mean ± standard error)**

**Day**	**Group A**	**Group B**	**Group C**	**Group D**	**Group E**
0	3.48 ± 0.27A(A)	3.37 ± 0.21A(A)	3.43 ± 0.22A(A)	3.45 ± 0.23A(A)	3.23 ± 0.21A(A)
7	0.37 ± 0.19B(B)	0.42 ± 0.17B(B)	0.78 ± 0.15C(B)	0.38 ± 0.15B(B)	3.37 ± 0.23A(A)
14	0.00 ± 0.00B(C)	0.00 ± 0.00B(C)	0.00 ± 0.00B(C)	0.00 ± .0.00B(C)	3.45 ± 0.23A(A)

Note: The different letters within a row denote significant differences between the difference groups (P < 0.05). The numbers in brackets represent the clinical therapeutic efficacy in accordance with the calculation of the decrease of the mites. The different letters in brackets within a column denote significant differences between the different treatment days (P < 0.05).

**Figure 1:**
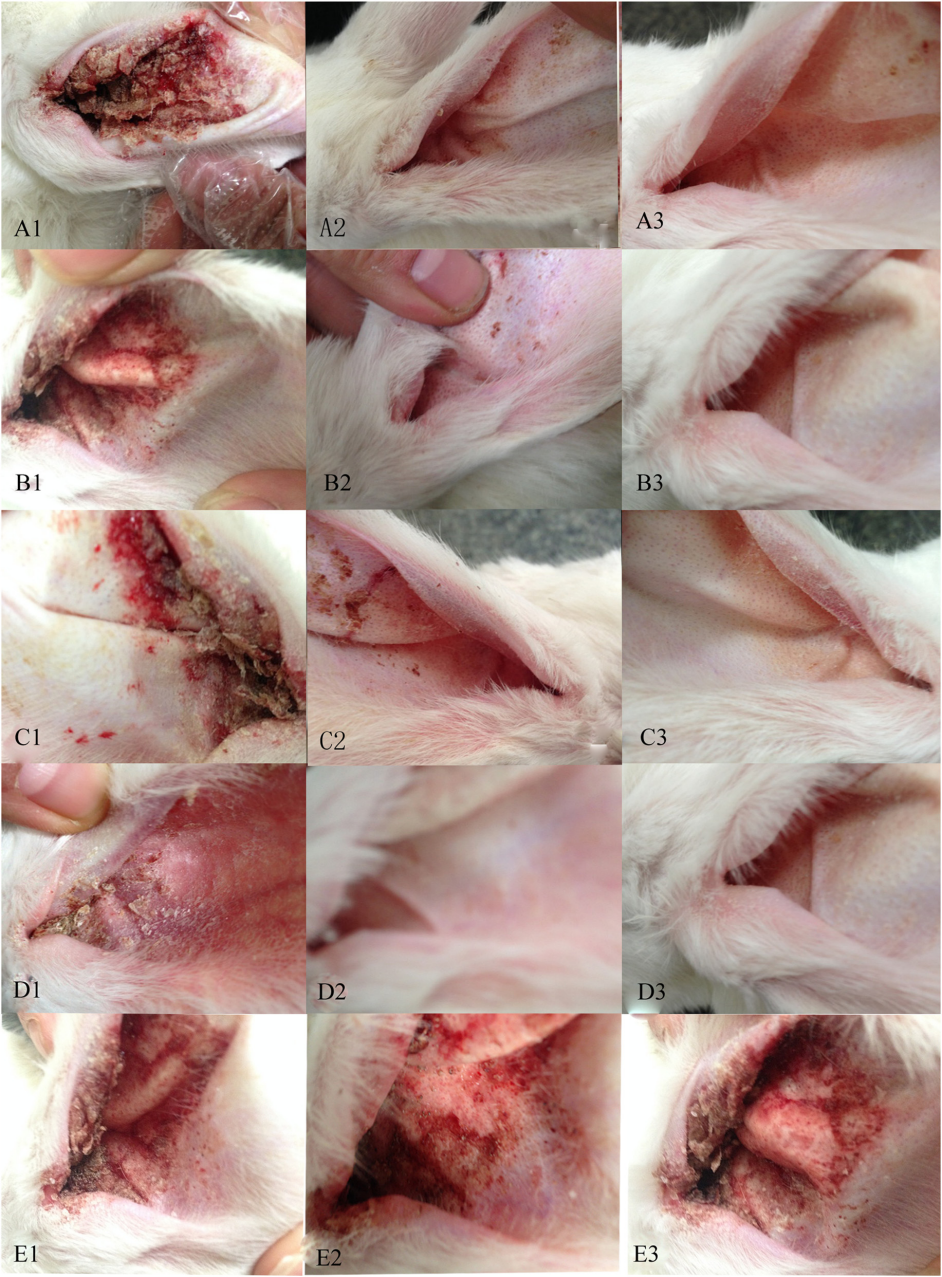
**Clinical observations of rabbits treated with euptox A against** ***P. cuniculi*** **. (A)**–**(E)** denote rabbits that were treated with euptox A at 4, 2, 1 mg/ml, ivermectin and glycerol and water only, respectively. A1, B1, C1, D1 and E1 show the signs of infection before treatment; A2, B2, C2, D1 and E1 show the observations at day 7; and A3, B3, C3, D1 and E1 show the observations at day 14.

All infected rabbits treated with 4, 2 and 1 mg/ml of euptox A (groups A, B and C) recovered and were completely cured by day 14 (Figure 1A3, B3 and C3). In addition, the rabbits in group A-C with 4, 2 and 1 mg/ml of euptox A displayed improved clinical signs during the experiment, although redness and inflammation were found in parts of the ear canals (Figure 1A2,B2 and C2). However, after the second treatment, the infected rabbits in group A-C were cured completely. Rabbits in group D (positive control) exhibited improvements in clinical signs during the experiment and no inflammation was observed (Figure 1D2 and D3). However, rabbits treated with negative control showed only minor signs of aggravated (Figure 1E2 and E3). After determining that there was no new crust formation after day 7, and that there were no mites on otoscopic examination, we treated scabs topically with the relevant euptox A (A, B and C groups) or ivermectin (D group).

Mites were collected and burdens assessed to provide values for mite reduction and negative transformation rate (%). From day 7, no mites were present in the left or right ears of any rabbits in these groups (A, B and D group). In group C, there were small amount of mites. Compared with the negative control group, treatment with euptox A gave significant improvements in clinical signs at each time point. The rabbit itching mite negative transformation rate in treatment group (A-D) were up to 100% 14 d after treatment (Table 3), and there were not badness (not irritant to the skin and toxicity).

**Table 3:** **The effects of euptox A on rabbit itching mite negative transformation rate**

**Time**	**0 d**	**7 d**	**14 d**	**Negative transformation rate %**
Group A	24.33 ± 1.97	0.00 ± 0.00	0.00 ± 0.00	100.00
Group B	21.50 ± 2.17	0.00 ± 0.00	0.00 ± 0.00	100.00
Group C	18.61 ± 2.17	2.17 ± 1.72	0.00 ± 0.00	100.00
Group D	24.61 ± 2.07	0.00 ± 0.00	0.00 ± 0.00	100.00
Group E	19.83 ± 2.13	22.83 ± 2.48	24.00 ± 2.37	−21.03

## Discussion

Good clinical efficacy was achieved with the euptox A extract from *E. adenophorum*, against the scab mites, *P. cuniculi*. The clinical acaricidal efficacy of euptox A was similar to that of injectable ivermectin, and this observation is consistent with the euptox A has strong toxicity against *S. scabiei* and *P. cuniculi in vitro* [26], whilst consistent with petroleum ether extract form *E.adenophorum* against *P. cuniculi in vitro* [31]*.* The clinical acaricidal efficacy was showed to be time- and concentration-dependent, with the euptox A displaying similar effects to the alcohol extract from *E. adenophorum* against *P. cuniculi*. In the current study, the euptox A showed better clinical acaricidal efficacy (2ml/ml) than the alcohol extract from *E. adenophorum* (1 g/ml) [32], whilst the petroleum ether extract of neem oil comparatively weak bioactivity (LC_50_, 500.0 ll/ml) [33].

*E. adenophorum* has been reported to have hepatotoxic effects in rodents [15,34,35] which could limit its development as an acaricidal agent. But the acute toxicity test and skin hypersensitive test of euptox A had been finished, the euptox A were not irritant to the skin and toxicity of rabbits. Combining our previous results with this trial, we firmly believe that, euptox A shows a good clinical therapeutic effect on animal acariasis.

## Conclusion

We believe that, after further in-depth study, euptox A, a potent herbal drug, will be more widely applied in treatments for humans and animals. This study provides a new way for utilization of *E. adenophorum* and the euptox A has the potential as acaricidal drugs in livestock, and future possible scope of the product in small animal vet medicine, e.g. against Octodectes ear mites and other common ectoparasites of dogs and cats. But in order to make the research systematic, the mechanism of action has yet to be determined.
